# Spinal anesthesia increases the risk of venous thromboembolism in total arthroplasty: Secondary analysis of a J-PSVT cohort study on anesthesia

**DOI:** 10.1097/MD.0000000000006748

**Published:** 2017-05-05

**Authors:** Mashio Nakamura, Masataka Kamei, Seiji Bito, Kiyoshi Migita, Shigeki Miyata, Kenji Kumagai, Isao Abe, Yasuaki Nakagawa, Yuichiro Nakayama, Masanobu Saito, Takaaki Tanaka, Satoru Motokawa

**Affiliations:** a Japanese National Hospital Organization—EBM Study Group, Japanese Study of Prevention and Actual Situation of Venous Thromboembolism after Total Arthroplasty (J-PSVT), Japanese National Hospital Organization; b Department of Cardiology and Nephrology, Mie University Graduate School of Medicine; c Center for Pulmonary Embolism and Venous Thrombosis, Murase Hospital; d Department of Clinical Anesthesiology, Mie University Graduate School of Medicine; e Division of Clinical Epidemiology, Japanese National Hospital Organization Tokyo Medical Center; f Department of Rheumatology, Fukushima Medical University; g Division of Transfusion Medicine, National Cerebral and Cardiovascular Center, Japan.

**Keywords:** arthroplasty, epidural anesthesia, general anesthesia, spinal anesthesia, venous thromboembolism

## Abstract

Clinical guidance on the choice of anesthetic modality vis-à-vis the risk of perioperative venous thromboembolism (VTE) is largely lacking because of a paucity of recent evidence. A comparative effect of general anesthesia and neuraxial blockade on the perioperative incidence of VTE has not been well-investigated.

We compared the effects of different types of anesthetic modalities on the risk of VTE after total hip arthroplasty (THA) and total knee arthroplasty (TKA).

This is a secondary analysis of the Japanese Study of Prevention and Actual Situation of Venous Thromboembolism after Total Arthroplasty (J-PSVT). Data pertaining to a total of 2162 patients who underwent THA and TKA at 34 hospitals were included in this analysis. We compared the different anesthetic modalities with respect to the incidence of VTE. The composite end-point was asymptomatic/symptomatic deep vein thrombosis detected using scheduled bilateral ultrasonography up to postoperative day (POD) 10 and fatal/non-fatal pulmonary embolism up to POD 10.

The study groups were as follows: general anesthesia (n = 646), combined epidural/general anesthesia (n = 1004), epidural anesthesia (n = 87), and spinal anesthesia (n = 425). On multivariate analysis, only spinal anesthesia was associated with a significant increase in the risk of VTE as compared with that associated with general anesthesia. Propensity score-matched analysis for “combined epidural/general anesthesia group” versus “spinal anesthesia group” demonstrated a 48% higher incidence of VTE (relative risk = 1.48, 95% confidence interval [CI] 1.18–1.85) in the latter.

Spinal anesthesia was associated with a higher risk of postoperative VTE, as compared with that associated with combined epidural/general anesthesia, in patients undergoing total arthroplasty.

## 1 Introduction

The association between venous thromboembolism (VTE) prophylaxis and anesthesia during orthopedic surgery has been well known for a long time. The association between the type of anesthesia and the risk of postoperative VTE is well documented. Vasodilation occurring during general anesthesia contributes to the onset of VTE by causing venous stasis, increase in venous capacitance, and decrease in venous return.^[1]^ Use of neuraxial blockade in orthopedic procedures has been shown to be safer in this respect with a low risk of VTE, intraoperative bleeding, length of hospital stay, and risk of surgical site infection.^[2,3]^ However, the advantage of neuraxial blockade with respect to VTE may no longer be relevant because of advances made in general anesthesia and pharmacological and mechanical thromboprophylaxis.^[4,5]^ Furthermore, extended neuraxial blockade, such as that required for epidural analgesia, may delay the initiation of postsurgical pharmacological prophylaxis owing to the associated risk of spinal and/or epidural hematoma, possibly leading to a permanent neurological deficit, such as paraplegia.^[6]^ There are no specific clinical guidelines for the choice of anesthesia, largely because of insufficient recent evidence from studies comparing general anesthesia and neuraxial blocks for their association with VTE.

Combined epidural/general anesthesia reduces blood loss and dosage requirement for anesthetic medication, is more effective in pain control, and causes fewer postoperative complications.^[7,8]^ Therefore, this anesthetic procedure has been widely used in orthopedic surgery for decades.^[9]^ The Japanese Study of Prevention and Actual Situation of Venous Thromboembolism after Total Arthroplasty (J-PSVT) is a multicenter, collaborative, prospective, observational study that investigates the efficacy and safety of VTE prophylaxis after joint replacement surgery, across 34 Japanese National Hospital Organization (NHO) hospitals. In the present study, we compared the effects of different types of anesthetic modalities on the incidence of VTE in patients undergoing total hip arthroplasty (THA) and total knee arthroplasty (TKA).

## 2 Methods

This was a secondary analysis of J-PSVT, a hospital-based, non-interventional, cohort study to assess the efficacy and safety of current practices for thromboprophylaxis. The design and main results of J-PSVT have been previously reported.^[10]^ Patients aged >20 years who were scheduled for THA or TKA for primary joint disease were eligible for inclusion. Data were collected from all patients undergoing primary THA and TKA between 2007 and 2010 in 34 NHO hospitals. The trial was registered in the Japan UMIN Clinical Trial Registry (UMIN000001366), and the study protocol was approved by the ethics committee of the NHO central institutional review board (No 0623004). Written informed consent was obtained from all patients prior to the use of their clinical records.

The study endpoint, which was a composite of asymptomatic/symptomatic deep vein thrombosis (DVT) up to postoperative day (POD) 10 and fatal/non-fatal pulmonary embolism (PE) up to POD 10, was compared with different types of anesthetic modalities.

All patients were assessed for DVT in the proximal and distal veins on POD 10 using standard compression ultrasonography (CUS). All sonographers were adequately trained. Just prior to the start of study, the sonographers received detailed instructions for standardized procedures in a specially organized conference herein they acquired the necessary certification. Symptomatic DVT and PE was diagnosed using CUS, contrast computed tomography, and lung perfusion scan.

Data on patient demographics, primary diagnosis, pre-existing comorbid conditions, length of operation, anesthesia type, and VTE prophylaxis were gathered using standard case report forms.

### 2.1 Statistical analysis

Discrete variables were compared using the chi-squared test; continuous variables were compared using Kruskal–Wallis rank test. Multiple logistic regression analysis was performed to identify the independent predictors of VTE. Variables with a *P* value of <.2 on univariate logistic regression analysis were included in a multivariate logistic regression model with stepwise forward selection, with forced entry of sex, surgery type, and each pharmacological prophylactic drug. These have been identified as risk factors for VTE in previous studies.^[4]^ Odds ratios (ORs) and 95% confidence intervals (CIs) were calculated as an approximation of the relative risk of VTE with different types of anesthetic modalities, taking general anesthesia as reference.

Propensity-score matching was performed to minimize the effects of confounding caused by non-randomized assignment to the type of anesthetic modalities. This was done to reduce the impact of selection bias and allow relevant variables to be evenly balanced in the combined epidural/general and spinal anesthesia groups. Tourniquet use was strongly associated with TKA because it was used in almost all patients undergoing TKA. Therefore, all variables except tourniquet use were included in the multiple logistic regression model to estimate the propensity score, which represents the probability of the use of either combined epidural/general or spinal anesthesia. A Markov chain Monte Carlo procedure was used for multiple imputation of missing values for numerical variables, such as body mass index and estimated glomerular filtration rate. A mode imputation procedure was used to impute missing values for categorical variables such as comorbidities. The multiple and mode imputation were performed using the XLSTATBase 2015 (Addinsoft, Paris, France). Patients undergoing combined epidural/general or spinal anesthesia were matched on a 1:1 basis using nearest-number matching using a caliper of 0.01. After matching, the incidence of postsurgical VTE was compared using the chi-squared test.

All reported *P* values are two-tailed. Data processing and analyses were performed using the Statistical Analysis System and IBM SPSS Statistics version 23.0 for Windows (SPSS, Chicago, IL).

## 3 Results

In total, 2186 patients met the inclusion criteria and were enrolled in the J-PSVT study. Primary effectiveness outcome was not assessed in 24 patients owing to the lack of ultrasonography or a preoperative history of antiplatelet therapy. Thus, the study population of this subgroup study consisted of 2162 patients. Based on the type of anesthesia, they were classified into the following groups: general anesthesia (n = 646), combined epidural/general anesthesia (n = 1004), epidural anesthesia (n = 87), and spinal anesthesia (n = 425) groups. The demographics and clinical characteristics of these patients are shown in Table 1.

**Table 1:**
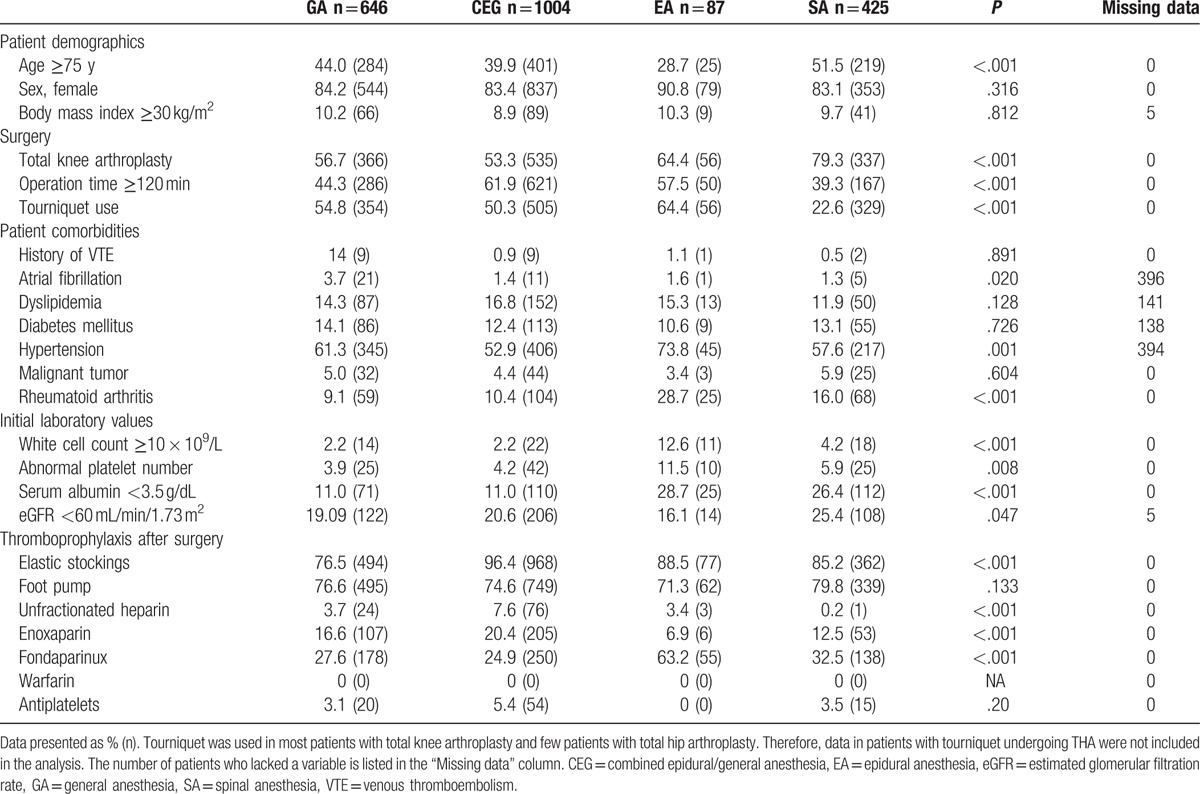
Baseline characteristics of the study group patients.

**Figure 1:**
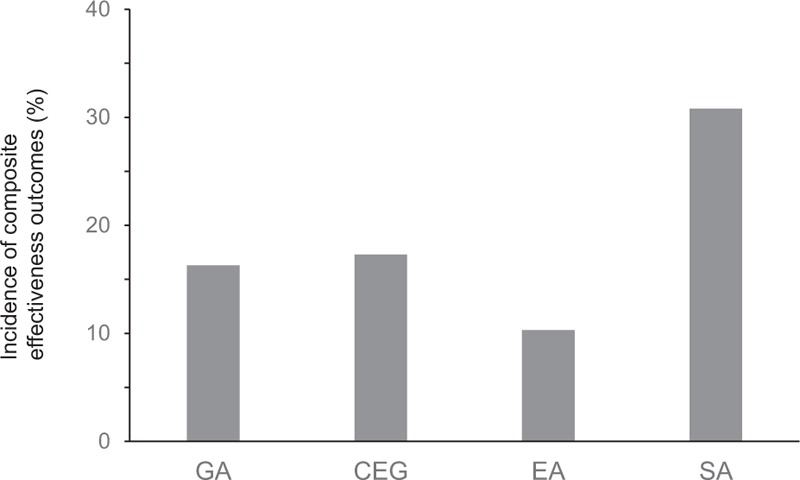
Incidence of venous thromboembolism in patients receiving different types of anesthetic modalities. Composite effectiveness outcomes were the results of asymptomatic/symptomatic deep vein thrombosis up to postoperative day 10 and fatal/non-fatal pulmonary embolism up to postoperative day 10. There was a significant difference in the incidence of venous thromboembolism between these 4 groups (*P* < .001). CEG = combined epidural/general anesthesia, EA = epidural anesthesia, GA = general anesthesia, SA = spinal anesthesia.

The incidence of VTE in the four groups was as follows: general anesthesia, 16.3%; combined epidural/general anesthesia, 17.3%; epidural anesthesia, 10.3%; and spinal anesthesia, 30.8% (Fig. 1). There was a significant difference in the incidence of VTE between these 4 groups (*P* < .001).

On multivariate analysis, only spinal anesthesia was significantly associated with an increased risk of VTE when compared with general anesthesia. Female sex, older age, and TKA were associated with increased VTE rates, which is consistent with an earlier report.^[4]^ Hypoalbuminemia and fondaparinux therapy was associated with a reduced incidence of VTE (Table 2).

**Table 2:**
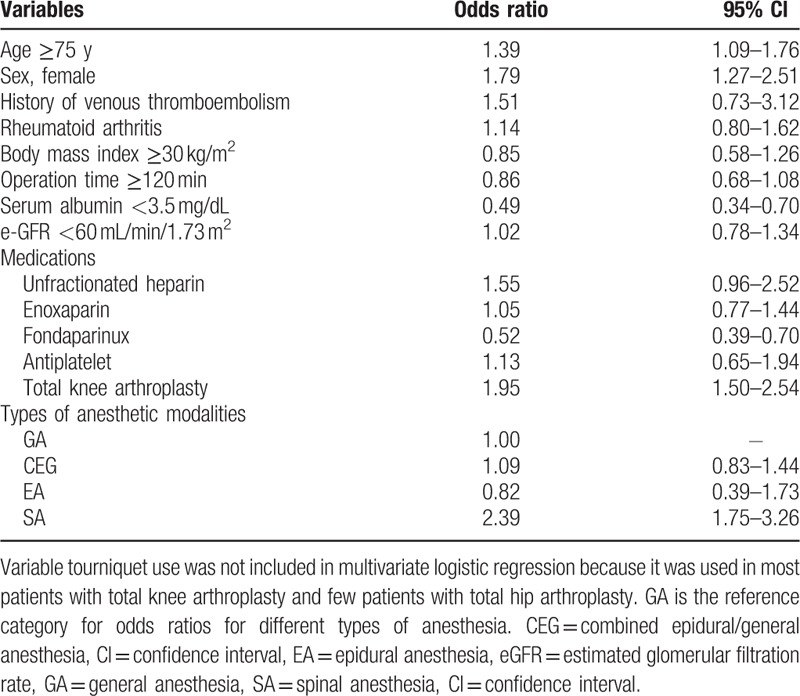
Results of multivariate logistic regression analysis for composite effective outcomes.

Finally, we directly compared combined epidural/general anesthesia and spinal anesthesia using propensity-score matching to minimize the confounding effects caused by non-randomized assignment to the type of anesthetic modalities. Concomitant liver and renal dysfunction and the use of antithrombotics may influence the choice in favor of neuraxial blockade. For this reason, the comparison of general anesthesia with spinal anesthesia is likely to be biased. Comparison of combined epidural/general anesthesia versus spinal anesthesia is likely to have eliminated this bias in our cohort. Moreover, the use of combined epidural/general anesthesia was the most frequent, whereas epidural anesthesia was the least frequent, in our cohort.

Propensity score matching allowed relevant variables to be balanced between the combined epidural/general anesthesia and spinal anesthesia groups, reducing the impact of selection bias. After propensity score matching, there were 305 patients in each group (Table 3).

**Table 3:**
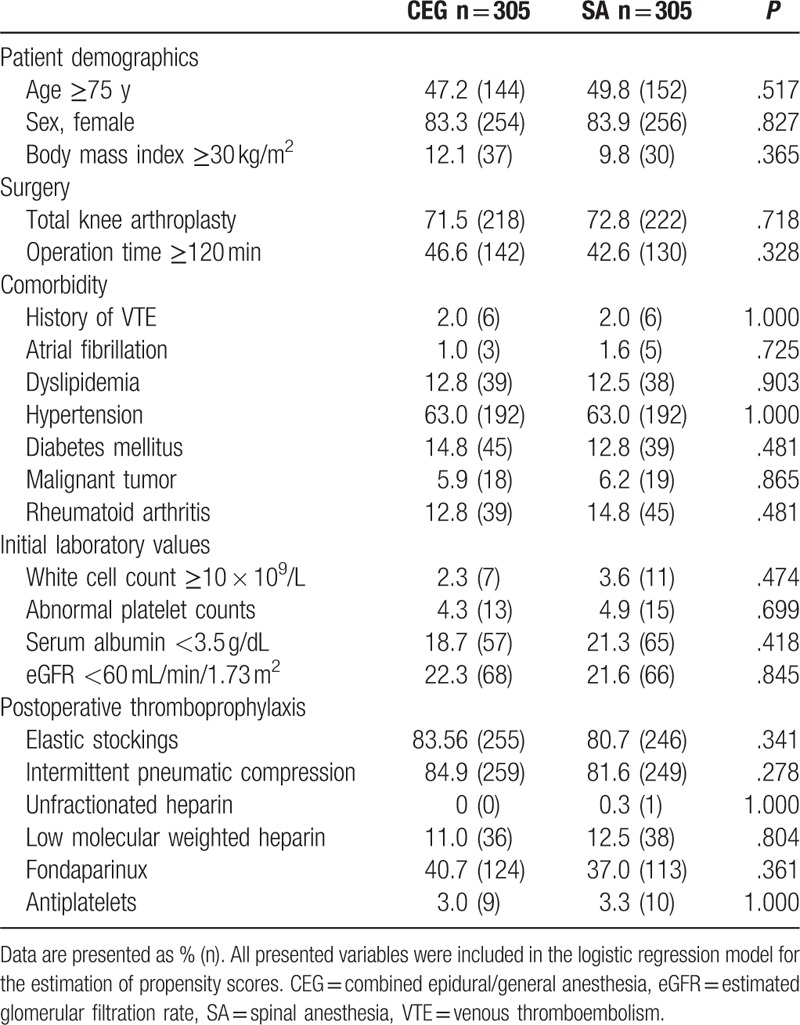
Baseline characteristics of propensity score-matched patients with combined epidural/general anesthesia and spinal anesthesia groups.

**Figure 2:**
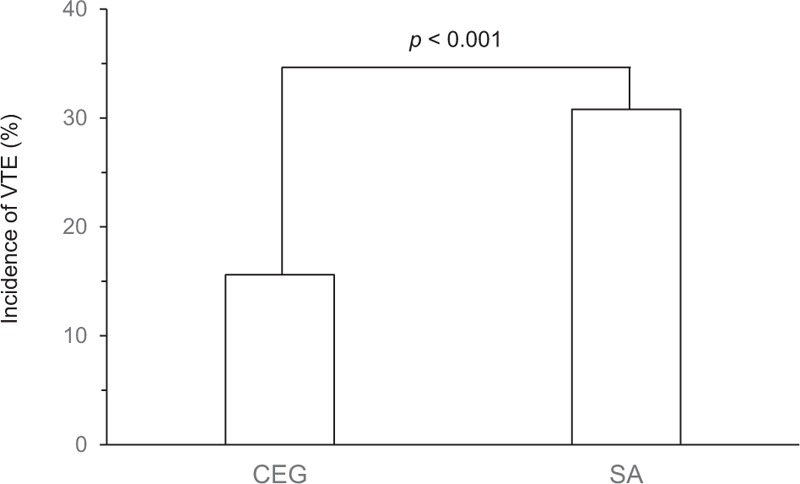
Incidence of venous thromboembolism among propensity-matched patients with combined epidural/general anesthesia and spinal anesthesia. White columns indicate the incidence of venous thromboembolism. There was a significant difference in the incidence of venous thromboembolism between 305 pairs of patients with combined epidural/general anesthesia and spinal anesthesia (*P* < .001). CEG = combined epidural/general anesthesia; SA = spinal anesthesia; VTE = venous thromboembolism.

In this study, spinal anesthesia increased VTE incidence by 48% (relative risk: 1.48, 95% CI, 1.18–1.85) when compared with that by combined epidural/general anesthesia (Fig. 2).

## 4 Discussion

In this study, spinal anesthesia was significantly associated with an increased risk of VTE in patients undergoing arthroplasty. On the other hand, VTE incidence did not differ between general anesthesia and combined epidural/general anesthesia. Although epidural anesthesia demonstrated a tendency for decreased VTE risk, this was not statistically significant owing to the relatively small sample size.

Neuraxial blockade is associated with a lower risk of VTE as compared with that associated with general anesthesia.^[11,12]^ Rheological changes in the hyperkinetic blood flow in the lower limbs may explain this effect. In addition, epidural anesthesia exerts a profibrinolytic action.^[13,14]^ Despite the good sensorimotor blockade, spinal anesthesia is associated with a higher incidence of hypotension.^[15,16]^ Majority of previous studies have compared epidural with general anesthesia; comparison between spinal and general anesthesia from the perspective of thromboprophylaxis in orthopedic surgery is not well-characterized.^[11,12,17]^ However, neuraxial blockade has little advantage when perioperative heparin prophylaxis is used.^[18–20]^ Indeed, pharmacological VTE prophylaxis is a standard component of the protocol of care for orthopedic patients.^[5]^

Conversely, epidural anesthesia added to general anesthesia has some advantages over general anesthesia, such as reduction in blood loss and the requirement for anesthetic medication.^[8]^ Moreover, combined epidural/general anesthesia markedly enhances venous flow in the leg because of its sympatholytic action; thus, minimizing perioperative venous stasis.^[21]^

However, neuraxial blockade, including spinal anesthesia, has not been compared with combined epidural/general anesthesia. Therefore, the mechanisms responsible for the effects of spinal anesthesia on postoperative thromboembolic events, relative to combined epidural/general anesthesia are unclear.

One reason for a high VTE incidence in patients with spinal anesthesia compared with those with epidural analgesia is the superior pain relief afforded by epidural analgesia, which facilitates the early postoperative mobilization of patients.^[22]^ The neuroendocrine response to clinical stress and/or surgery is associated with increased hemostasis.^[23]^ Other reasons may be that spinal anesthesia produces stronger freezing in the leg as compared with that by combined epidural/general anesthesia, whereas the outcome may vary according to the anesthetic agent used in epidural anesthesia.^[24]^ However, from a hematological perspective, there were no differences in perioperative hemostatic markers between general and spinal anesthesia.^[19]^ Continuous epidural analgesia in the postoperative recovery period attenuated some markers of hypercoagulability.^[25]^

Our study has several limitations. First, we consider DVT for lower leg to be an unsuitable endpoint for multicenter studies owing to the limitation of large inter-observer differences. Second, detailed information on some anesthesia-related parameters such as, level of postoperative analgesia and the timing of epidural catheter removal was not included in the analysis. Third, we did not include data on in–out balance (blood/solution infusion vs. discharge volume), blood pressure, and cardiac output during anesthesia.

Despite these limitations, our data may provide useful information on the choice of anesthesia. Surgeons who perform THA and TKA for patients at high VTE risk may consider avoiding spinal anesthesia, although careful risk and benefit assessment of spinal anesthesia should be conducted. Further studies, such as randomized controlled trials, are warranted to confirm our results.

In conclusion, our study suggests that spinal anesthesia may be associated with an increased risk of postoperative VTE, as compared with that associated with combined epidural/general anesthesia, in patients undergoing THA and TKA.
